# Transoral Approach to Parapharyngeal Space Tumours: Preliminary Reports from a Single-Centre Retrospective Analysis

**DOI:** 10.3390/curroncol30040297

**Published:** 2023-03-30

**Authors:** Giovanni Motta, Domenico Testa, Anna Donadio, Filippo Ricciardiello, Michele Cavaliere, Eva Aurora Massimilla, Gaetano Motta

**Affiliations:** 1 ENT Unit-Department of Mental, Physical Health and Preventive Medicine, University of Campania “Luigi Vanvitelli”, 80131 Naples, Italy; 2 Ear, Nose and Throat Unit, AORN “Antonio Cardarelli”, 80131 Naples, Italy; 3 Otorhinolaryngology-Head and Neck Surgery Unit, Department of Neuroscience, Reproductive and Odontostomatological Sciences, University of Naples Federico II, 80138 Naples, Italy

**Keywords:** transoral approach, parapharyngeal space, benign tumours

## Abstract

Purpose: The aim of this study is to identify certain parapharyngeal space tumours with specific characteristics that can be treated successfully through an endoscopically assisted transoral approach (EATA). Methods: Nine patients with PPS tumours underwent surgery through an EATA between 2003 and 2021. All patients underwent clinical examination and fibrolaryngoscopy. Preoperative CT and/or MRI was performed on all patients. Results: All the patients were successfully treated through an endoscopically assisted transoral approach. Histological examination revealed five pleomorphic adenomas, two schwannomas, one ectopic thyroid gland and one lipoma. The only long-term sequelae observed was Horner syndrome in the two schwannomas arising from the carotid space. The mean hospitalisation time was 2.6 days, while the mean follow-up time was of 9.7 years. Conclusions: An endoscopically assisted transoral approach (EATA) is a valid technique for treating benign capsulated tumours of the true PPS and some benign capsulated tumours of the superomedial aspect of the carotid space.

## 1. Introduction

The PPS (PPS) is a fascial space of the suprahyoid neck that contains fat and neurovascular structures, and it is surrounded by other fascial spaces. The PPS is described as an inverted pyramid, whose base is represented by the skull base and the apex by the superior cornu of the hyoid bone. The antero–medial border is represented by the superior constrictor of the pharynx, while laterally, it is demarcated by the medial pterygoid muscle, by the deep lobe of the parotid gland and by the mandible ramus. Posteriorly, it is demarcated by the prevertebral fascia that separates the PPS from the retropharyngeal space. The pre-styloid compartment is occupied by fat and by the deep lobe of the parotid gland, while the retro-styloid PPS is occupied by neurovascular structures such as the internal jugular vein, the internal carotid artery, cranial nerves IX to XII, the cervical sympathetic chain, fat and lymph nodes [1,2]. However, the nomenclature of the PPS has changed: what used to be known as pre-styloid space is now recognised as the true PPS, while the post-styloid space is identified as the carotid space.

PPS tumours are extremely rare, representing approximately 0.5% of all the neoplasms involving the head and neck district [1]. About 80% of PPS lesions are benign and have a salivary gland origin [2]. A wide variety of neoplasms can arise from this space, but salivary gland tumours, followed by neurogenic tumours, are recognised as the most frequent [3].

The surgical approach to the PPS must be tailored to the nature, dimensions and site of the tumour. There are a range of surgical techniques used to treat PPS tumours, with the transcervical approach being the most frequently used [3,4,5].

Amongst the various surgical approaches, the transoral approach has been widely criticised compared to the transcervical approach. This has been mainly attributed to the inadequate exposure of the approach, which restricts tumour visualisation, leading to increased risk of neurovascular injury and a higher risk of tumour spillage and recurrence over time [6]. However, various authors suggest that the transoral approach is a valid surgical technique for certain tumours of both the true PPS and the carotid space [7,8]. Similarly, other authors describe the transoral approach as a safe method for removing benign tumours of the superomedial aspect of the PPS, with decreased morbidity and shorter hospitalisation time compared to transcervical approaches [9,10].

In this retrospective single-centre study, we describe the feasibility of an endoscopically assisted transoral approach (EATA) for the treatment of certain PPS tumours.

## 2. Materials and Methods

Nine patients underwent parapharyngeal space surgery through an endoscopically assisted transoral approach (EATA) between January 2003 and October 2021 at the ENT Unit of Vanvitelli University. Of the nine patients enrolled in this study, seven were female (77.7%) and two were male (22.2%), with an age range of 67–28 years old and a mean age of 48.5. After a careful clinical intraoral examination, and after evaluation of the patients by fibreoptic endoscopic examination, a computer tomography (CT) scan and/or magnetic resonance imaging (MRI) were performed in order to obtain an optimal anatomical characterisation of the parapharyngeal lesions. For a correct surgical planning of each case, we have heavily relied on imaging techniques such as CT and/or MRI. In all cases, the clinical examination showed prominent masses that caused oropharyngeal bulging. These masses were not palpable in the neck. In all cases, there was no evidence of suspicious lymph nodes during neck palpation. Imaging techniques confirmed the clinical examination and highlighted, in all cases, the presence of a capsule that contained the PPS tumour.

**Figure 1:**
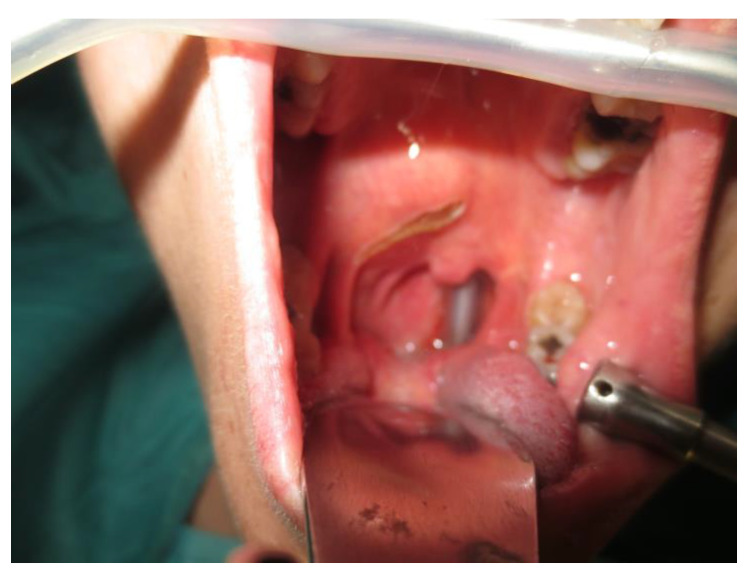
Incision.

**Figure 2:**
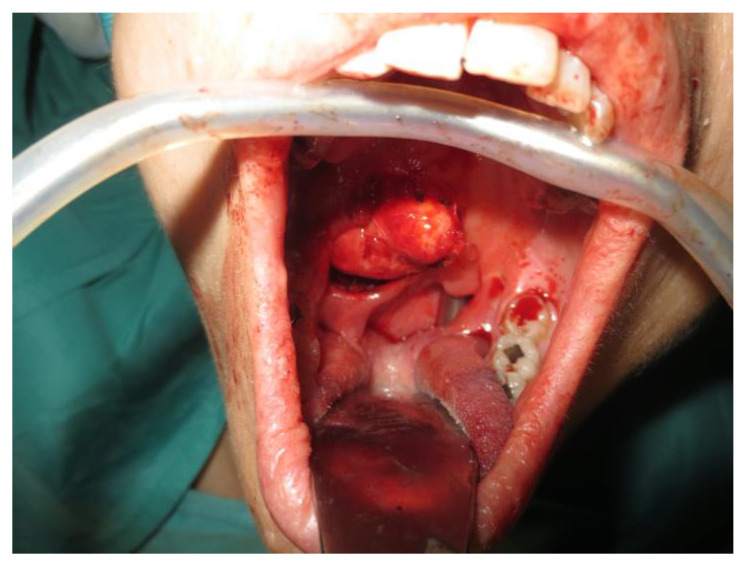
Exposure of the PPS tumour.

**Figure 3:**
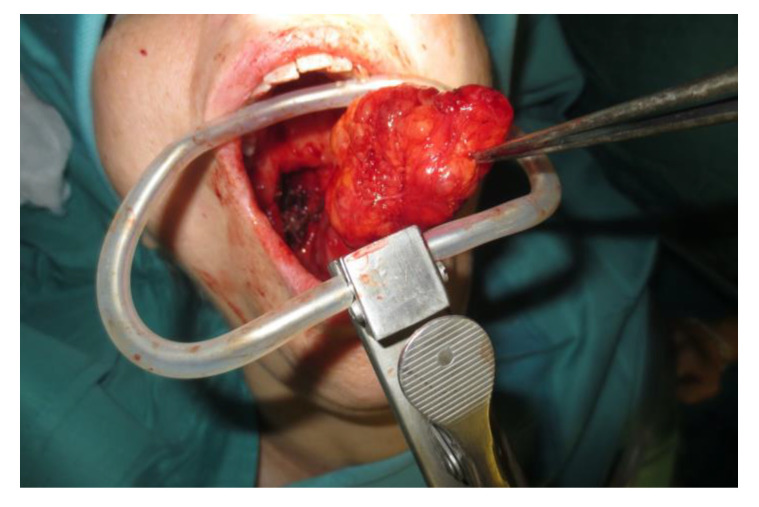
En bloc removal of the PPS tumour.

**Figure 4:**
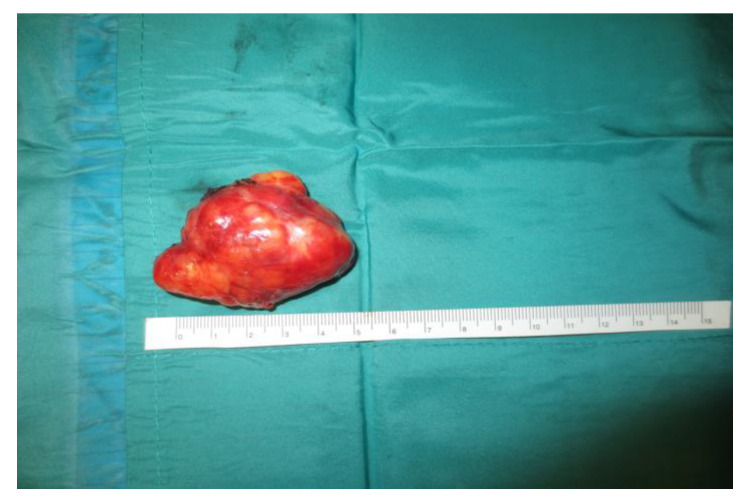
Tumour size and macroscopical features.

Operative technique: Using an electrosurgical knife, an incision is carried out at the level of the anterior pillar of the palatine tonsil (Figure 1). The incision can be extended superiorly to the soft palate or inferiorly towards the base of the tongue, according to the necessities of the surgical case. The dissection is continued in an antero–posterior direction through the superior constrictor muscle of the pharynx, gaining access to the PPS and exposing the tumour (Figure 2). Starting from the anterior aspect of the tumour, a blunt dissection is then performed. Endoscopic assistance is used for checking (0°, 30° and 45° endoscope 4 mm in diameter), facilitating the dissection of other aspects of the tumour that cannot be clearly seen under direct view; it also enables the visualisation of all the important landmarks in the PPS. Once these structures are identified, it is possible to remove the endoscope and proceed with a finger dissection for the en bloc removal of the tumour using the tumour capsule as a cleavage plan (Figure 3). After the removal of the tumour from the PPS, accurate haemostasis is obtained. In order to achieve careful haemostasis, the visualization of the surgical field is optimized using the endoscope. The surgical wound is then closed with 2.0 absorbable vicryl stitches. The macroscopical characteristics and the tumour size of one of the tumours of this casuistry (histologically diagnosed as pleomorphic adenoma) are shown below (Figure 4).

**Ethical considerations:** all patients had given their informed consent and a Research Protocol was approved by the Ethical Committee of our University Department. The study was conducted in accordance with the Helsinki Declaration.

## 3. Results

**Figure 5:**
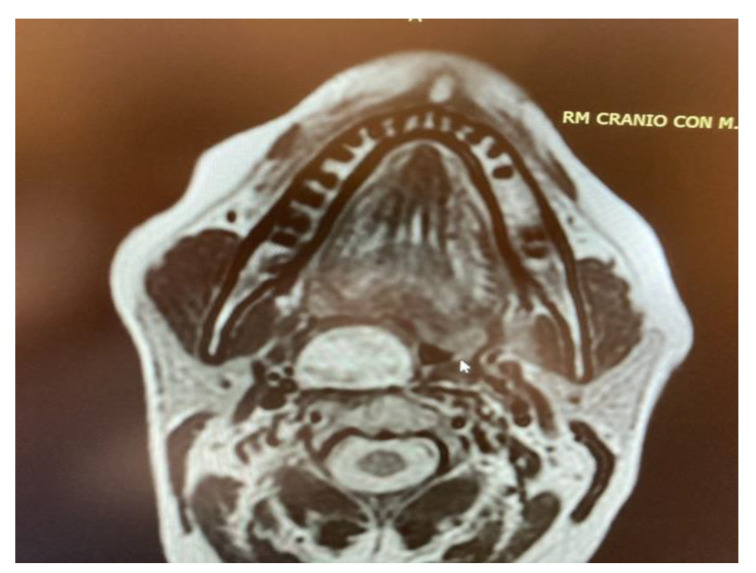
MRI T2 weighted image showing a right parapharyngeal schwannoma arising from the cervical sympathetic chain. The schwannoma does not dislocate the big vessels in the typical anterior direction, but the big vessels’ dislocation is posterolateral. However, the parapharyngeal fat pad is dislocated in an anterior direction, and this suggests a carotid space tumour.

**Figure 6:**
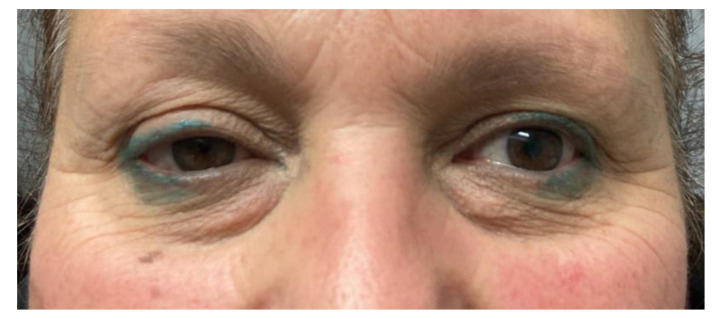
Horner syndrome.

Nine patients with PPS tumours were successfully treated through an endoscopically assisted transoral approach (EATA). In all cases, the oropharyngeal masses were not palpable at the level of the neck, and no suspicious lymph node was detected during the clinical examination. Only two patients out of nine were asymptomatic (22.2%), while the other seven patients complained of symptoms such as dysphagia (66.6%), foreign body sensation (55.5%), dyspnoea (11.1%) and dysphonia (11.1%). The histology and the site of the PPS tumours are reported below. All of these tumours were capsulated:

1. **Five pleomorphic adenomas** that arose from minor salivary glands in three cases and from the deep lobe of the parotid gland in two cases. All of these tumours were situated in the true PPS, medially with respect to the great vessels. In all the patients, no complications were observed during and after surgery. Hospitalisation time was two days for three patients, three days for one patient and four days for the other patient. No tumour recurrence was observed after sixteen, nine, eight and seven years for the first four patients. Evaluating the recurrence in the last patient is still not possible due to the short follow-up period, since the patient underwent surgery in 2021.
2. **One lipoma** arose from the true parapharyngeal space. The patient underwent surgery in 2012 and had no intraoperative and no postoperative complications. No recurrence was observed after ten years. The hospitalisation time was two days.
3. **One ectopic thyroid** arose from the right true parapharyngeal space. This was the only case in which a fine needle aspiration citology (FNAC) was performed, and the results were inconclusive. The patient underwent surgery in 2003 with no intraoperative or postoperative complications. The hospitalisation time was three days. No recurrence observed after eighteen years.
4. **Two schwannomas** (Figure 5) originating from the cervical sympathetic chain and that arose in the superomedial aspect of the PPS, medially to the carotid sheath. The first patient underwent surgery in 2008, the second in 2019. No complications were observed during surgery and no tumour recurrence has been observed after 14 and 4 years, respectively. The only long-term complication observed was Horner syndrome (Figure 6), despite the continuity of the nerve not being interrupted. The hospitalisation time was three days for both patients.

The mean surgery time was 53.3 min. The mean hospitalisation time was 2.6 days and the mean follow-up time was 9.7 years. The mean follow-up time for pleomorphic adenoma patients treated through a transoral approach was 8.2 years. When leaving the hospital, a liquid and soft diet was administered to the patients for ten days. These results are summarised in Table 1.

## 4. Discussion

Tumours in the PPS are uncommon, representing approximately 0.5% of all head and neck neoplasms; about 80% are benign and 20% are malignant [11,12]. Prestyloid tumours are more common (59%) than retrostyloid tumours (26%) [13]. Salivary gland tumours account for 40–50% of cases [3,14,15,16]. These tumours are located in the prestyloid space and can arise from either the deep lobe of the parotid gland or from minor salivary glands. Lopez [3] reports that fewer than 5% of parotid tumours involve the PPS. A fat plane on CT or MRI between the mass and the parotid helps distinguish a tumour of minor salivary origin from a deep lobe parotid tumour [3,6]. Fortunately, in most cases, salivary gland tumours of the PPS have a benign origin (80%) with a histological diagnosis of pleomorphic adenoma [3,16], followed by other benign neoplasms such as monomorphic adenoma, Warthin tumour and oncocytoma. Instead, amongst the malignant salivary gland tumours of the PPS (20%), the most common histotypes are the mucoepidermoid carcinoma, carcinoma ex pleomorphic adenoma and the adenoid cystic carcinoma [3].

Amongst the various PPS tumours considered in this study, all of them were of a benign nature and pleomorphic adenoma was the most commonly represented histotype (55.6%). Histological examination revealed that the other four cases were two schwannomas (22.2%), one ectopic thyroid gland (11.1%) and one lipoma (11.1%). Of these nine neoplasms, seven of them (77.8%) were located in the true parapharyngeal space and the other two (22.2%) were located in the carotid space.

The signs and symptoms of PPS neoplasms can be subtle and clinical evaluation of this space is difficult. A large proportion of patients have asymptomatic growth for a long period of time, and the tumour is often detected during scans that are being performed for other reasons [3,17]. When present, the most common symptoms include dysphagia and foreign body sensation [16], and this was also found to be the case in our study sample, as these were the most common symptoms referred to by the patients. This is due, as in our cases, to the tumour, which can cause oropharyngeal bulging which compromises the deglutitory capacity of the patients [1,16]. Many approaches have been described to remove PPS tumours; their excision can be difficult because of the deep location, complex anatomy and the surrounding vital structures [6,11,12].

An appropriate approach should allow wide enough exposure of the tumour to enable complete resection and manage the complications, with minimal aesthetic and functional morbidity [3,18]. It is essential to preserve the integrity of the capsule (or pseudocapsule) to avoid tumour spillage and to avoid multifocal recurrence in the case of surgically approaching pleomorphic adenomas [19]. All available approaches have advantages and drawbacks [6,11,12,20]. The transoral approach is the most controversial due to its narrow access, which restricts tumour visualisation and thus increases the risks of injury to the major neurovascular bundle, tumour spillage and uncontrolled haemorrhage.

However, this approach has many advantages, including a low risk of injury to the facial and inferior alveolar nerves [6,10,12] and the development of salivary fistulae, no need for an external incision (scarring is avoided), no major complications requiring mandibular osteotomy and a shorter hospital stay, minimal blood loss and reduced postoperative pain compared to other approaches [3,10].

The endoscopic transoral approach (EATA) has the advantages of adequate illumination and a direct operative field with a possibility of tactile feedback, enabling efficient and safe minimal invasive surgery. Endoscopy provides excellent visualization, allowing the verification of adequate haemostasis and the absence of residual tumour [3,7].

Numerous authors have reported positive results using this technique for the transoral removal of PPS tumours [7,21,22,23,24,25] but obviously the patients who might be potential candidates for a transoral surgical approach must be carefully selected [6,7,10,12].

The rate of nondiagnostic FNAC in PPT is moderately high (25–60%) as a result of excessive bleeding, lack of cellular material and other technical problems related to adequately targeting the lesion in close proximity to major neck vessels. A biopsy would be more accurate in the diagnosis, and it is usually performed when a malignant tumour is suspected. However, there could be a risk of tumour spillage in cases of pleomorphic adenoma [3,13,26,27]. Only one of our patients underwent a pre-operative bioptic procedure, with inconclusive results.

Imaging exams such as CT, and especially MRI, are necessary for the choice of the correct surgical approach, providing important information regarding the relationship of the tumour to surrounding structures, including the internal carotid artery, the internal jugular vein, the parotid gland, cranial nerves IX–XII and the distance from the skull base. Based on their variegated nature, MRI is crucial for highlighting the different features of PPS tumours: Pleomorphic adenomas are hypointense in T1 and hyperintense in T2. Schwannomas are isointense or hypointense in T1, with high enhancement with gadolinium, and hyperintense in T2, just like pleomorphic adenomas, from which they differ due to the different sites of origin and different patterns of fat and vessels dislocation. Other neurogenic tumours of the carotid space that need to be identified and distinguished from schwannomas are paragangliomas: These tumours have the characteristic “salt and pepper” aspect on T2 weighted images due to the presence of flow voids. Flow voids are indicators of a highly vascularised mass so that, in tumours with these features, a transoral approach is contraindicated due to the high risk of uncontrollable and unmanageable haemorrhage [3]. Importantly, it is possible to determine, using imaging exams, the site of origin and the potential nature of a PPS tumour, evaluating the direction of the parapharyngeal fat pad dislocation [3,6]. Parotid gland tumours tend to dislocate the parapharyngeal fat pad anteromedially; masticator space tumours tend to dislocate it posteromedially; carotid space tumours tend to dislocate it anteriorly and pharyngeal tumours dislocate it posterolaterally. The PPS fat pad is dislocated by PPS tumours, as are the great vessels that pass through this space, with anteromedial displacement of the carotid sheath structures in carotid space tumours and posterolateral displacement of the carotid sheath structures in tumours arising from the true PPS [3,6].

In accordance with the previously cited authors [3,5,16], pleomorphic adenoma was also found to be the most frequent histotype in this study. These tumours arise in the real PPS and are capsulated focal tumours, so a transoral approach is feasible in cases when the tumour itself displays oropharyngeal bulging. The transoral approach would not be feasible in the event of multifocal pleomorphic adenoma. However, the incidence of multiple primary multicentric pleomorphic adenoma is extremely rare in patients with no prior history of trauma or surgery, which is why it may be beneficial to adopt minimal margin surgery using the tumour capsule as a dissection plane, especially when using the nerve monitoring systems available today [19].

**Figure 7:**
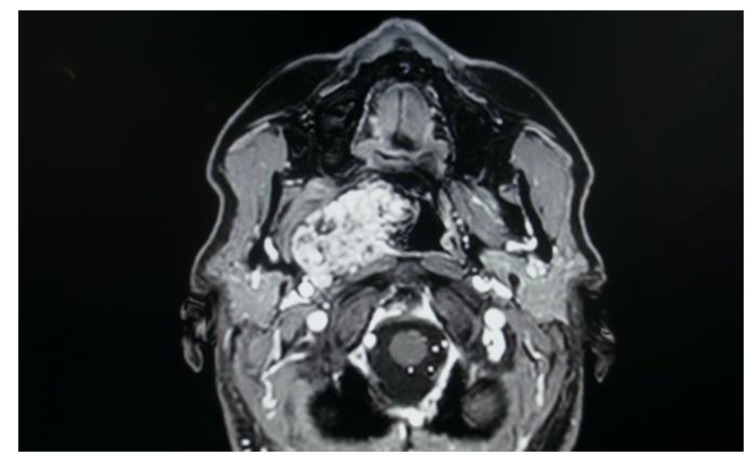
Pleomorphic adenoma of the true PPS originating from extraparotid salivary tissue.

In the study sample, imaging exams are evaluated for correct and safe surgical planning of each case. As shown in the MRI imaging, the parapharyngeal pleomorphic adenoma originated from the true parapharyngeal space with anteromedial dislocation of the parapharyngeal fat pad and posterolateral dislocation of the carotid sheath structures. The radiological evidence of a linear fat line between the tumour and the parotid gland confirmed that the tumour did not originate from the deep lobe of the parotid gland, but from extraparotid accessory salivary tissue (Figure 7). Two pleomorphic adenomas originated from the deep lobe of the parotid gland and, in these cases, MRI showed the absence of a linear fat line between the tumour and the deep lobe of the parotid gland.

**Figure 8:**
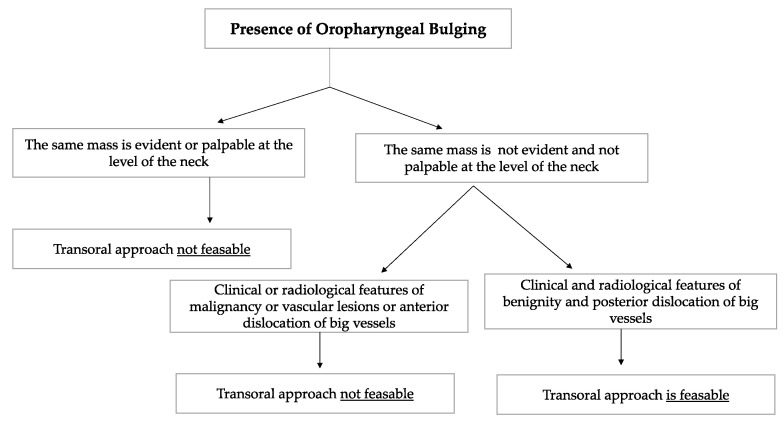
The flow chart shows the criteria considered when deciding whether or not to opt for a transoral approach to the PPS. Among the clinical and radiological features of benignity, the absence of the oropharyngeal mass at the level of the neck, the absence of suspicious lymph nodes during neck palpation, radiological confirmation of the clinician neck examination and the presence of a capsule that contained the PPS tumour were considered. The transoral approach was considered infeasible in vascular tumours and in tumours that gave anterior dislocation of the big vessels.

Schwannomas are the most frequent neurogenic tumours involving the parapharyngeal space. In the PPS, schwannomas can originate either from the vagus nerve or from the cervical sympathetic chain [3,6]. They rarely originate from the hypoglossal nerve [3]. Vagal schwannomas are usually located beneath the hyoid bone, while sympathetic chain schwannomas are more commonly located above it [28]. Their radiological appearance is peculiar: vagal schwannomas split the internal jugular vein from the internal carotid artery, leading to an increase in the distance between the artery, which is dislocated anteriorly, and the vein, which is dislocated posteriorly. In such schwannomas, a transoral approach would be contraindicated due to the big vessels dislocation pattern, which increases the risk of vascular injury and unmanageable haemorrhage. On the other hand, these great vessels are not separated by cervical sympathetic chain schwannomas [28]. Since they originate from the carotid space, anterior displacement of the great vessels is more common [6,25,28]. In such cases, which represent the great majority of cervical sympathetic chain schwannomas, a transoral approach is not feasible in our opinion due to the position of the great vessels, which increases the risk of vascular injury. A flow chart presenting the criteria taken into consideration in order to opt for a transoral approach is shown in Figure 8.

However, carotid space tumours do not always lead to anterior displacement of the great vessels. It is possible, as demonstrated by the schwannoma cases presented herein, that cervical sympathetic schwannomas may lead posterior displacement of the great vessels, just as parapharyngeal tumours arising from the true PPS [29]. In these cases, it might be helpful to look at parapharyngeal fat pad dislocation, which in carotid space tumours is anterior, even when the vessels are dislocated posteriorly (Figure 5). Due to the suprahyoid location, the superomedial position of the tumours and the posterolateral dislocation of the internal carotid artery, a transoral approach was preferred in both cases. Larger, clearly circumscribed tumours, even those involving the carotid space, can be removed transorally with an acceptable safety profile, but only if the big vessels are dislocated posteriorly [7,30]. This approach provided a good visualisation for the en bloc resection of both tumours and better exposure than the one achieved by a transcervical technique. After surgical removal of these tumours, Horner syndrome is frequently observed as an inevitable sequela due to the specific nervous origin of the tumour, even when an intracapsular dissection is performed [31] (Figure 6).

Chen [6] reports in his 12 cases, one case of hoarseness in a vagus nerve schwannoma and one case of Horner’s syndrome in cervical sympathetic chain schwannoma. No complication occurred in 10 pleomorphic adenomas.

The other transoral technique used to gain access to the PPS is the use of transoral robotic surgery (TORS). TORS has recently been attempted for the treatment of PPS tumours [32,33]. TORS is a feasible and safe approach for the removal of certain PPS tumours, but has some technical drawbacks [3,7,30,32]. Robotic dissection does not provide surgeons with tactile feedback, and this may well explain the higher rate of capsule rupture in case of pleomorphic adenoma during a transoral robotic dissection [30,32]. It is likewise undeniable that the robot is a relatively new technique, few studies are available and the follow-up time is still too short to establish if the recurrence rate of the PPS tumours treated with TORS is acceptable [32,33,34].

**Figure 9:**
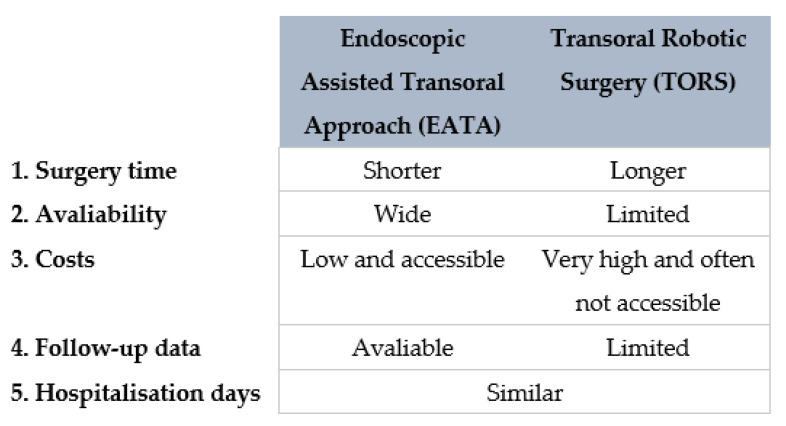
Diagram comparing EATA and TORS.

Moreover, the robot is not widely available, is extremely expensive and involves longer operations [7,30]. A resumptive comparing the two transoral techniques is shown in Figure 9.

In conclusion, careful clinical evaluation, with intraoral examination and neck palpation, combined with imaging exams such as CT and especially MRI, are extremely important and can often prove sufficient for establishing which PPS tumour can be treated using a transoral approach. In our experience, a transoral approach is suitable not only for true PPS capsulated tumours located medially to the great vessels, but also for certain carotid space capsulated tumours that involve the superomedial aspect of the PPS, which are located medially to the carotid sheath and lead to posterior dislocation of the internal carotid artery. The presence of a capsule, providing surgeons with tactile feedback, helps to avoid vascular damage during transoral dissection, even when the tumour is located in the superomedial aspect of the carotid space. Moreover, in the latter situation, the transcervical approach would not be ideal due to the poor access of this approach to the medial and superior aspects of the PPS [7,8].

Compared with the traditional transoral approach, the EATA has the advantages of adequate illumination offering the view of a wide and direct operative field, enabling efficient and safe minimally invasive surgery. Further studies are required to evaluate the feasibility and the effectiveness of the intraoral techniques.

### Limitations of the Study

This study displays several limitations. Firstly, the small size of the cohort is the main weakness of this work; on the other hand, this study was only intended to present a preliminary case series. Secondly, the retrospective nature of the study could represent a bias, given the lack of some data. Thirdly, this work only reported PPS tumours treated using EATA and lacks data about other surgical approaches.

## 5. Conclusions

According to our preliminary experience, the EATA to PPS tumours is a feasible surgical technique for benign capsulated tumours of the true PPS, and for some benign capsulated tumours of the superomedial aspect of the carotid space with posterolateral dislocation of the internal carotid artery and without extension to the skull base. We believe that in capsulated tumours of the true PPS which display oropharyngeal bulging, and in some superomedial capsulated tumour of the carotid space with atypical great vessels displacement, an intraoral approach is far more appropriate than external approaches, since the parapharyngeal space is the deepest space of the neck. The advantages of this approach include avoidance of an external scar and short hospitalisation times. Further studies are required to clarify the advantages and disadvantages of such an approach to treating PPS tumours.

## Figures and Tables

**Table 1:** Resumptive table.

Age	Sex	Signs	Symptoms	Location	Surgery Time	Intraoperative Complications	Tumour Size (cm)	Histological Diagnosis	Hospitalization Days	Post-Operative Sequalae	Follow Up (Years)	Recurrence
86	Male	Oropharyngeal mass	Dyspnea, dysphagia	True PPS	60 min	None	4×3×5.2	Benign thyroid tissue	3	None	19	No
75	Female	Oropharyngeal mass	Dysphagia	Carotid space	70 min	None	3×4×3	Schwannoma	3	Horner Syndrome	14	No
53	Male	Oropharyngeal mass	Asymptomatic	True PPS	20 min	None	4×2.5×3	Lipoma	2	None	10	No
56	Female	Oropharyngeal mass	Dysphagia, Foreign body sensation	True PPS	40 min	None	5×3.3×4.2	Pleomorphic Adenoma	2	None	9	No
59	Female	Oropharyngeal mass	Dysphagia, Foreign body sensation	True PPS	50 min	None	5×3×3.3	Pleomorphic Adenoma	2	None	7	No
57	Female	Oropharyngeal mass, snoring	Dysphagia, Foreign body sensation	Carotid space	120 min	None	2.6×2.6×2.2	Schwannoma	3	Horner Syndrome	4	No
49	Female	Oropharyngeal mass	Dysphagia, dysphonia Foreign body sensation	True PPS	50 min	None	6×3.4×4.5	Pleomorphic Adenoma	3	None	1	No
55	Female	Oropharyngeal mass	Foreign body sensation,	True PPS	40 min	None	4×2×2	Pleomorphic Adenoma	2	None	16	No
36	Female	Oropharyngeal mass	Asymptomatic	True PPS	30 min	None	3×2×2.2	Pleomorphic Adenoma	4	None	8	No

## Data Availability

Data are available upon reasonable request.
